# Control of Uniaxial Negative Thermal Expansion in Layered Perovskites by Tuning Layer Thickness

**DOI:** 10.3389/fchem.2018.00455

**Published:** 2018-10-18

**Authors:** Chris Ablitt, Arash A. Mostofi, Nicholas C. Bristowe, Mark S. Senn

**Affiliations:** ^1^Department of Materials and The Thomas Young Centre, Imperial College London, London, United Kingdom; ^2^Department of Physics, Imperial College London, London, United Kingdom; ^3^School of Physical Sciences, University of Kent, Canterbury, United Kingdom; ^4^Department of Chemistry, University of Warwick, Coventry, United Kingdom

**Keywords:** NTE, perovskite, Ruddlesden-Popper, anisotropy, compliance, corkscrew, thermal expansion

## Abstract

Uniaxial negative thermal expansion (NTE) is known to occur in low *n* members of the A_*n*+1_B_*n*_O_3*n*+1_ Ruddlesden–Popper (RP) layered perovskite series with a frozen rotation of BO_6_ octahedra about the layering axis. Previous work has shown that this NTE arises due to the combined effects of a close proximity to a transition to a competing phase, so called “symmetry trapping”, and highly anisotropic elastic compliance specific to the symmetry of the NTE phase. We extend this analysis to the broader RP family (*n* = 1, 2, 3, 4, …, ∞), demonstrating that by changing the fraction of layer interface in the structure (i.e., the value of 1/*n*) one may control the anisotropic compliance that is necessary for the pronounced uniaxial NTE observed in these systems. More detailed analysis of how the components of the compliance matrix develop with 1/*n* allows us to identify different regimes, linking enhancements in compliance between these regimes to the crystallographic degrees of freedom in the structure. We further discuss how the perovskite layer thickness affects the frequencies of soft zone boundary modes with large negative Grüneisen parameters, associated with the aforementioned phase transition, that constitute the thermodynamic driving force for NTE. This new insight complements our previous work—showing that chemical control may be used to switch from positive to negative thermal expansion in these systems—since it makes the layer thickness, *n*, an additional design parameter that may be used to engineer layered perovskites with tuneable thermal expansion. In these respects, we predict that, with appropriate chemical substitution, the *n* = 1 phase will be the system in which the most pronounced NTE could be achieved.

## 1. Introduction

**Figure 1:**
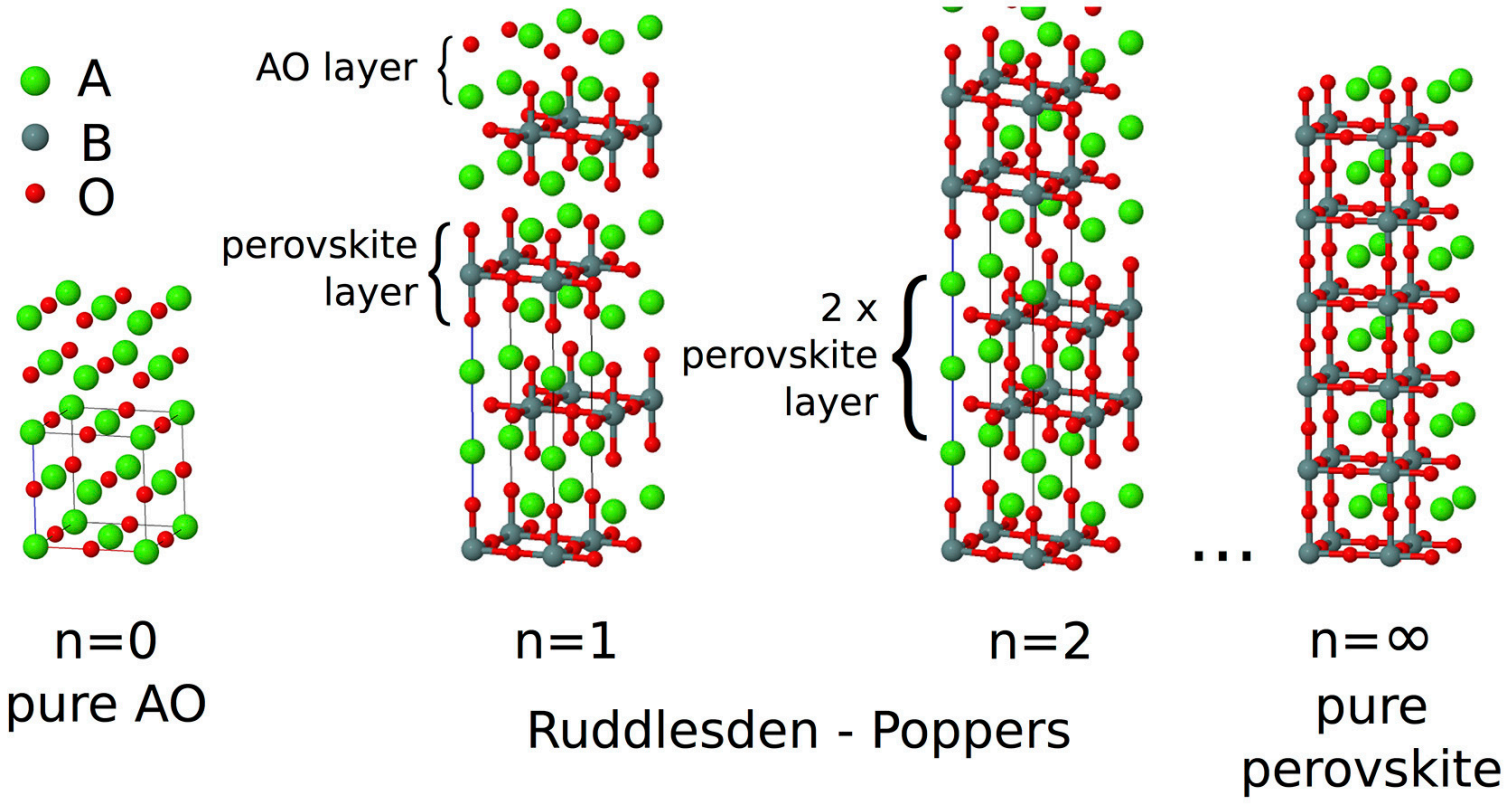
In the A_*n*+1_B_*n*_O_3*n*+1_ Ruddlesden–Popper series, blocks consisting of *n* layers of ABO_3_ perovskite structure are separated by a single layer of AO rock salt structure, with BO_6_ octahedra in the next block displaced by a (0.5, 0.5, 0.5) lattice translation. In the *n* = ∞ limit the pure ABO_3_ perovskite structure is recovered.

Ruddlesden–Popper (RP) oxides are an intriguing class of ceramic materials. They have the basic formula A_*n*+1_B_*n*_O_3*n*+1_ and consist of a perovskite block of *n* corner sharing BO_6_ octahedra separated by an AO rock salt layer. Blocks of octahedra are stacked perpendicular to the long crystallographic axis making this layering axis structurally distinct from the two in-plane axes. Neighboring blocks are de-phased from each other by a lattice translation of (0.5, 0.5, 0.5), and the aristotypical symmetry for any value of *n* is *I*4/*mmm* (Figure 1). As with the perovskites, the A-site chemistry is dominated by larger alkali, alkali-earth, and rare earth metals, and the B-site by transition metals. In the limit *n* = ∞, the perovskite structure is recovered. While in practice most chemistries are found to predominantly exhibit the *n* = 1, 2 phases only (Palgrave et al., 2012), in principle any value of *n* between 1 and ∞ is possible; *n* = 3 structures have been synthesized by careful compositional control (Battle et al., 1998) and although *n* > 3 phases are often predicted to be unstable to decomposition (McCoy et al., 1997), epitaxial growth techniques have allowed the synthesis of *n* = 2−5 (Haeni et al., 2001), *n* = 6 (Yan et al., 2011), and *n* = 10 (Lee et al., 2013) structures.

One of the most explored systems is the *n* = 1 A_2_CuO_4_ on account of its high-temperature superconductivity, where doping of divalent A = Ba and Sr with trivalent rare earth cations has been extensively investigated (Dwivedi and Cormack, 1991). Superconductivity in these systems is not limited to the cuprates, and there has been substantial interest in Sr_2_RuO_4_ (Mackenzie and Maeno, 2003) on account of its superconducting phase transition below 0.8 K, and in Sr_3_Ru_2_O_7_ for its nematic orbitally-ordered phase (Borzi et al., 2007). The doped nickelates have also been much studied due to their believed proximity to a superconducting phase transition, and their charge ordering physics (Yoshizawa et al., 2000). More recently, the *n* = 2 member of the RP family has received much attention on the account of a new form of improper ferroelectricity predicated in Ca_3_Mn_2_O_7_ and Ca_3_Ti_2_O_7_, termed hybrid improper ferroelectricity (Benedek and Fennie, 2011). This mechanism circumvents the so-called *d*^0^ criterion for ferroelectricity, as it does not require an off-centring of cations to drive the phase transition. Instead, this off-centring (*P*) may occur as a slave process driven by an octahedral tilt (*R*_1_) and rotation mode (*R*_2_) of the parent structure that are inherently unstable in some of these systems. This leads to a so-called trilinear term β*R*_1_*R*_2_*P* in the free energy expansion about the parent structure (Benedek et al., 2015) which, regardless of the sign of the coefficient β, as *R*_1_ and *R*_2_ are inherently unstable, leads to a non-zero value of the polarization *P* (either positive or negative depending on the sign of β).

Our contribution to this field of hybrid improper ferroelectricity was to provide experimental confirmation of this mechanism for the case of Ca_3_Ti_2_O_7_ (Senn et al., 2015). However, our high-resolution powder diffraction data for Ca_3_Mn_2_O_7_ revealed an added complexity. What was believed to be a single phase at room temperature, having the polar symmetry *A*2_1_*am*, was in fact a mixture of this and a phase that we identified as having *Acaa* symmetry. Crucially, this phase only has a single octahedral rotation that is out-of-phase rather than in-phase, and the octahedra remain untilted along the *c*-axis. No hybrid improper ferroelectric mechanism is therefore possible. However, over the large phase coexistence region, which spans a temperature range of 120 K, we did observe pronounced uniaxial negative thermal expansion (NTE) along the *c*-axis in the *Acaa* phase. This had not been observed previously in the *n* = 2 system and, although reported in the literature in an analogous *n* = 1 system (Takahashi and Kamegashira, 1993), its significance had not been noted. We were able to explain this NTE phenomenon as being driven by a leftover degree of freedom, the octahedral tilt in the *Acaa* phase, which remains dynamic.

NTE is a rare property, that when it does occur is known to be caused by a diverse range of mechanisms in different materials. Even within inorganic perovskite-based systems, NTE has been found to originate due to coupling of the lattice parameters to: charge ordering (Azuma et al., 2011), ferroelectric ordering (Chen et al., 2013), and magnetic and orbital ordering via an invar-like mechanism (Yoshida et al., 2005, Qi et al., 2010). In framework structures, formed from connected strongly-bonded polyhedral units, NTE has been explained by transverse vibrations of these units, known as rigid-unit modes (RUMs) (Dove et al., 1995, Heine et al., 1999). We argued in *Acaa* Ca_3_Mn_2_O_7_ that certain vibrational modes with RUM character would have negative Grüneisen parameters and be soft on account of the proximity of the system to the symmetry-forbidden phase transition to *A*2_1_*am* (Senn et al., 2015). Using this idea of “trapping” a soft mode in the *Acaa* phase of Ca_3_Mn_2_O_7_ to systematically control and tune the uniaxial thermal expansion properties of the solid solution Ca_3−*x*_Sr_*x*_Mn_2_O_7_ (Senn et al., 2016), we were able to demonstrate that this is a property exclusively of the *Acaa* phase in these materials, and that NTE is enhanced as the system approaches the *A*2_1_*am* phase boundary as a function of chemical composition *x*. Although other effects operate in related materials, in this study we restrict our discussion to NTE driven by the coupling of the cell parameters to soft lattice modes since it is the most appropriate mechanism to describe our system.

The presence of dynamic octahedral tilts in this Ca_3−*x*_Sr_*x*_Mn_2_O_7_ system explained the thermodynamic driving force for NTE along the layering axis. However, the question remained open of why NTE was only observed in this *Acaa* phase with a frozen in-plane rotation and not in the high-symmetry *I*4/*mmm* phase or related ABO_3_ perovskite phases, where dynamic octahedral tilts would still operate. We were able to answer this question in a recent computational study using density functional theory (DFT) and working within the quasi-harmonic approximation (QHA) to reproduce experimentally measured uniaxial NTE in the *I*4_1_/*acd* phase of *n* = 1 Ca_2_MnO_4_ (Ablitt et al., 2017).

**Figure 2:**
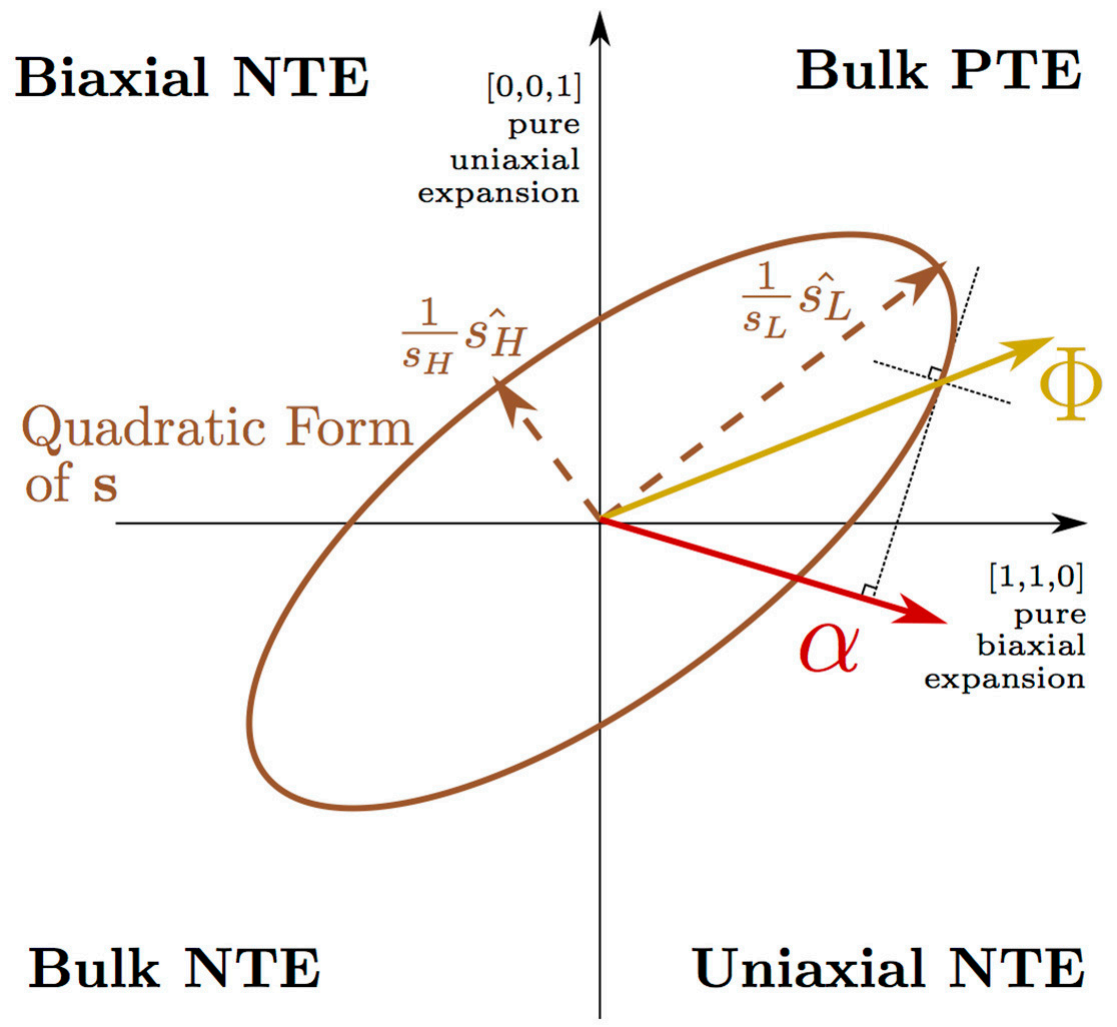
Equation (1) is illustrated on axes describing normal cell deformations using Voigt notation (e.g., where the vector [ϵ, ϵ, 0] corresponds to normal strains of ϵ of the *a* and *b* axes with the *c* axis unstrained). A 3 × 3 elastic compliance matrix ***s*** is shown here in its quadratic form as an ellipsoid projected onto the ($\bar{1},1,0$) plane. ***s*** may transform a vector describing the thermodynamic driving force for thermal expansion, **Φ**, in the positive quadrant (corresponding to bulk positive thermal expansion), into a thermal expansion vector, **α**, in the quadrant corresponding to uniaxial negative thermal expansion of the *c* axis. The direction of **α** is given in the figure by utilizing the radius normal property of the representation ellipsoid of the matrix ***s***.

Equation (1) (Grüneisen and Goens, 1924) describes the thermal expansion, α_η_(*T*), at temperature, *T*, of the three cell axes of a tetragonal crystal (η = 1, 2, 3 where α_1_ = α_2_ by symmetry). Equation (1) is explained in detail in Appendix 1 and the concept of a Φ vector driving bulk PTE being transformed by a highly anisotropic ***s*** into uniaxial NTE (Barron and Munn, 1967) is depicted pictorially in Figure 2. In this picture, the anisotropic thermal expansion is separated into a thermodynamic driving force vector, Φ—arising from the lattice dynamics—that is transformed by the anisotropic elastic compliance matrix, ***s***. By computing the compliance matrix for our NTE phase, we were able to extract the thermodynamic driving force vector from our QHA simulation and found that the effect from dynamic tilts alone would not predict NTE over the wide temperature range observed in experiment. It is only when this thermodynamic driving force is transformed by the highly anisotropic elastic compliance of the layered RP phase that our simulations demonstrated uniaxial NTE of a magnitude and over a temperature range comparable to experiment. Comparing the compliance matrices computed for different phases, we found that particularly high anisotropic compliance is unique to the NTE phase of the RP structure and we linked this anisotropy to combined in-plane (frozen rotations) and out-of-plane (the AO layer) symmetry breaking in the NTE phase.

(1) $$\begin{array}{l} \left(\begin{array}{c} \alpha_{1}\left(T\right)\\ \alpha_{1}\left(T\right)\\ \alpha_{3}\left(T\right) \end{array}\right)=\left(\begin{array}{ccc} s_{11} & s_{12} & s_{13}\\ s_{12} & s_{22} & s_{13}\\ s_{13} & s_{13} & s_{33} \end{array}\right)\left(\begin{array}{c} \Phi_{1}\left(T\right)\\ \Phi_{1}\left(T\right)\\ \Phi_{3}\left(T\right) \end{array}\right) \end{array}$$

Until now, our research has focused on understanding uniaxial NTE in the low *n* RP compounds. In the present paper, we focus on extending these concepts to predict how the material properties that we have linked to NTE develop as a function of *n*. Studying the Ca_*n*+1_Ge_*n*_O_3*n*+1_ system within the framework of DFT, we find that the magnitude of anisotropic elastic compliance is dependent upon the proportion of CaGeO_3_:CaO interface in the structure—which may be conveniently expressed by the fraction 1/*n*. This high compliance is then maximized with the highest proportion of interface (*n* = 1). To provide an explanation for this key result, we analyze how the components of the compliance matrix vary with 1/*n*. We identify a series of regimes in which groups of structures display similar elastic behavior based on the atomic degrees of freedom allowed by symmetry, and propose mechanisms by which these internal degrees of freedom couple to cell strains. The most important of these is the atomic “corkscrew” mechanism that operates at this interface in the NTE phase. We go on to investigate how the frequencies of the softest (lowest frequency) octahedral tilt modes, which provide the thermodynamic driving force for NTE, vary as a function of *n* in the high-symmetry and NTE phases. We find that a higher proportion of interface causes these phonons to stiffen (increase in frequency) and we infer that within a given chemical composition, the layer thickness *n* provides a structural constraint on an approximate temperature window for which the NTE phase will be stable—where increasing *n* drives this window to higher temperatures. One may thus use this insight, combined with our previous discovery that chemical substitution within a given structure may be used to tune the thermodynamic driving force for NTE, to use layer thickness *n* and composition as design parameters to engineer RP phases with optimal thermal expansion properties.

The layout of the paper is as follows: section 2 gives details of simulation parameters used in this work; section 3 is then split into four subsections: section 3.1 presents the key result of the paper, showing how the magnitude of anisotropic compliance, linked with uniaxial NTE, varies with 1/*n*; for the more interested reader, sections 3.2 and 3.3 then take a step back and analyze the origin of the result in section 3.1 in more detail, in section 3.2 by analyzing how the different elements of the compliance matrix evolve with 1/*n* and in section 3.3 by identifying different compliance regimes linked to crystallographic degrees of freedom in the structure that couple to strain; finally in section 3.4 we consider how the thermodynamic driving force for NTE varies with 1/*n* by presenting lattice dynamics calculations investigating phonons corresponding to tilts of GeO_6_ octahedra in Ca_*n*+1_Ge_*n*_O_3*n*+1_, and compare these results against experimental phase diagrams constructed with data taken from the literature of the analogous Ca_*n*+1_Mn_*n*_O_3*n*+1_ system. Additionally, in the associated Appendices file, Appendix 1 gives a brief overview of the mathematical concepts relevant for the study of thermal expansion in an anisotropic material and Appendix 2 outlines the symmetry of the phases simulated throughout this study.

## 2. Methods

Calculations were performed using CASTEP, a plane-wave DFT code, version 7.0.3 (Clark et al., 2005). A plane wave cut-off energy of 1,400 eV was employed for all calculations with electron density stored on a grid twice as dense. A 7 × 7 × 2 Monkhorst–Pack grid of kpoints shifted away from the Γ-point was used for calculations of the 14 atom *I*4/*mmm* phase of (*n* = 1) Ca_2_GeO_4_, with grids of equivalent reciprocal space density used for all other structures (high-symmetry and rotation phases for *n* = 1, 2, 3, 4, ∞—see Appendix 2). Norm-conserving pseudopotentials, generated on-the-fly using CASTEP version 16.0, were used for all calculations and the associated pseudopotential strings may be found Table S1 in the Supplementary Information. All calculations used the PBEsol exchange-correlation functional (Perdew et al., 2008). Absolute energies were converged to an accuracy of 0.5 meV/atom with respect to k-point grid density and plane wave cut-off energy. Geometric relaxations were performed with a force tolerance of 10^−4^ eV/Å and a stress tolerance of 10 MPa.

We expect our Ca_*n*+1_Ge_*n*_O_3*n*+1_ system to be well-described by conventional GGA density functionals. There are other members of the chemical space that might require more careful consideration in terms of the appropriate methodology, such as hybrid functionals, DFT+U, or potentially even DMFT in order to accurately describe the physics associated with localized d and f-electrons.

Elastic constants were computed by fitting second order polynomials to the energies of cells with applied strains of ±0.2, 0.4% from the fully relaxed cell, where the internal degrees of freedom (the atomic positions) were free to relax. The quadratic terms to these fits were used to construct terms within the elastic constant matrix, **c**, and this matrix inverted to compute the elastic compliance matrix, ***s*** (see Appendix 1 for the definition of ***s*** studied).

Bulk moduli, *K*, were computed by allowing the cell and all internal degrees of freedom to relax in response to hydrostatic pressures in the range −2 to +2 GPa. The bulk modulus was then found by fitting the computed relaxed volume, *V*, as a function of the external pressure, *P*, to the equation: $K=-\frac{d\left(ln\left[V\right]\right)}{dP}$. The bulk compressibility, β, is then given by β = *K*^−1^.

Density functional perturbation theory (DFPT) was used within CASTEP (Refson et al., 2006) to perform phonon calculations. In the present study, only phonon frequencies computed at single, high symmetry q-points are reported. In *n* = 1, 3, ∞ phases, these are at the *X* (½, ½, 0) and *P* (½, ½, ½) points in the *I*4/*mmm* high-symmetry phase (labeled *M* and *R*, respectively, in *n* = ∞ $Pm\bar{3}m$ ABO_3_) and for *n* = 2, 4 phases at the *X*-point in *I*4/*mmm*. Phonons in child rotation phases were always computed at the Γ-point. In every compound studied, the initial structure was the highest symmetry phase that was fully relaxed. All child phases were found by freezing unstable phonons (modes with imaginary frequencies) into the structure with small amplitudes and allowing this child structure to relax. The lattice parameters and cell energies relative to the high-symmetry parent of all relaxed structures may be found in Table S2.

## 3. Results and discussion

### 3.1. Anisotropy of the compliance matrix

**Figure 3:**
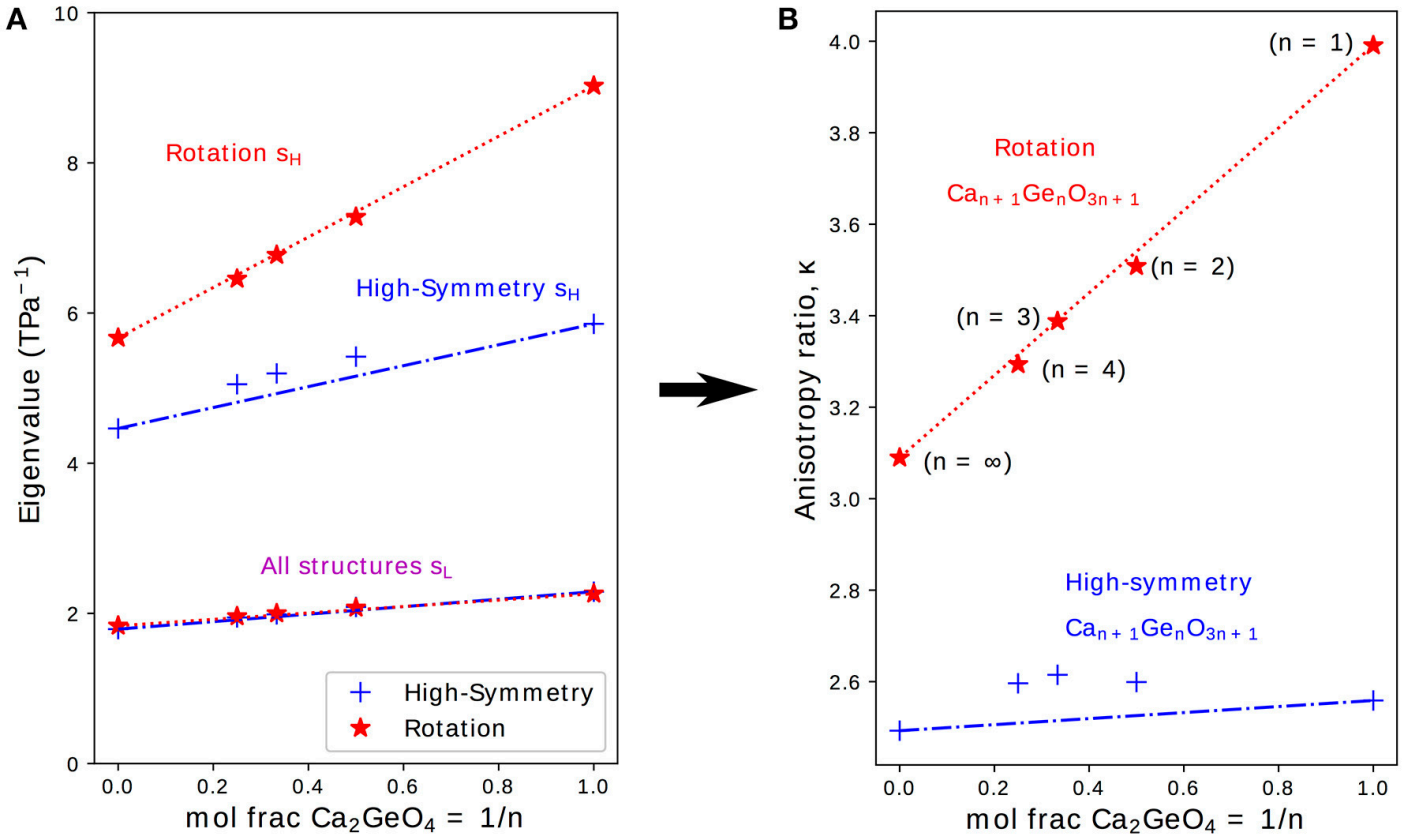
**(A)** Lowest, *s*_*L*_ and highest, *s*_*H*_, eigenvalues to the compliance matrix, ***s***, and **(B)** the anisotropy ratio κ plotted for high-symmetry and rotation phases in the Ca_*n*+1_Ge_*n*_O_3*n*+1_ series against the mole fraction of CaGeO_3_:CaO interface (1/*n*). Interpolations between values in the CaGeO_3_ (1/*n* = 0) and Ca_2_GeO_4_ (1/*n* = 1) structures are plotted for both high-symmetry and rotation phases (blue dot-dashed and red dotted lines, respectively).

It was previously shown in first-principles calculations performed on the NTE phase of Ca_2_GeO_4_ (i.e., the *I*4_1_/*acd* rotation phase) that a highly anisotropic ***s*** is an essential ingredient for uniaxial NTE in this system (Ablitt et al., 2017). κ, as defined in Equation (2), is the ratio of the highest (*s*_*H*_) and lowest (*s*_*L*_) eigenvalues of ***s*** and gives a measure of the anisotropy of ***s***; where higher κ indicates that ***s*** is more conducive to uniaxial NTE. Figure 3 therefore shows how *s*_*H*_, *s*_*L*_, and κ evolve with varying *n* for high-symmetry and rotation phases in the Ca_*n*+1_Ge_*n*_O_3*n*+1_ series.

(2) $$\begin{array}{l} \kappa =\frac{s_{H}}{s_{L}} \end{array}$$

In the composite mechanics community, the elastic properties of laminates are typically described by the properties of the constituent phases, weighted by the relative fraction of that phase (Sarlosi and Bocko, 2016). However, in the functional oxides community, it is well known that the local structure of atomic interfaces within a crystal often dictate the physical properties of the entire material 2012. There are therefore two ways to conceive the layered RP structure shown in Figure 1: (i) as being comprised of constituent CaGeO_3_ and CaO phases or (ii) as being comprised of CaGeO_3_ and the CaGeO_3_:CaO interface.

(3) $$\begin{array}{l} {\text{Ca}}_{n+1}{\text{Ge}}_{n}{\text{O}}_{3n+1}={\left[{\text{CaGeO}}_{3}\right]}_{n}\left[\text{CaO}\right]\\ ={\left[{\text{CaGeO}}_{3}\right]}_{n-1}\left[{\text{CaGeO}}_{3}:\text{CaO}\right] \end{array}$$

From Equation (3), it is clear that (i) the mole fraction of CaO in Ca_*n*+1_Ge_*n*_O_3*n*+1_ is given by the ratio 1/(*n* + 1) and (ii) the mole fraction of Ca_2_GeO_4_, which represents the fraction of CaGeO_3_:CaO interface in the structure, is given by the ratio 1/*n*. Therefore, to reflect the importance of the interface, *s*_*H*_, *s*_*L*_, and κ are plotted as a function of 1/*n* in the subplots on Figure 3. Straight lines have also been plotted interpolating between values for the CaGeO_3_ (1/*n* = 0) and Ca_2_GeO_4_ (1/*n* = 1) end members to show how well the structure may be considered as a mixture of these two constituents in the high-symmetry (blue dot-dashed) and rotation (red dotted) phase series.

The least compliant eigenvector, *s*_*L*_, corresponds to isotropic expansion/contraction for all structures investigated (see Table S3) and thus is closely linked to the bulk compressibility, β. Figure 3 shows that *s*_*L*_ increases linearly with higher Ca_2_GeO_4_ mole fraction but is invariant to changes in symmetry for a given *n*. All values for *s*_*L*_ lie on the line interpolating between CaGeO_3_ and Ca_2_GeO_4_ high-symmetry end members regardless of phase symmetry implying that *s*_*L*_ is determined mainly by the composition.

*s*_*H*_ also increases in magnitude with Ca_2_GeO_4_ content for all RP phases. However, unlike *s*_*L*_, *s*_*H*_ is greatly enhanced in the phase with a frozen rotation compared to the high-symmetry phase, and the rate of increase in *s*_*H*_ for rotation phases with 1/*n* is also greater in the rotation phase than in the high-symmetry parent. For all the tetragonal phases studied, the eigenvector *s*_*H*_ lies in a strain direction corresponding to a cooperative increase in in-plane lattice parameters, *ab*, and decrease in the lattice parameter along the layering axis, *c*, or visa versa. In a previous work, we proposed an atomic mechanism to facilitate a large compliance eigenvector in RP phases with a frozen octahedral rotation that relies on combined in-plane and out-of-plane symmetry breaking at the CaGeO_3_:CaO layer interface to closely couple the *ab* and *c* axes (Ablitt et al., 2017). Since this mechanism operates at the CaGeO_3_:CaO interface, it is interesting to note that *s*_*H*_ in the rotation phase is linearly dependent upon the mole fraction of this interface in the structure, increasing as this interface fraction becomes greater, and thus *s*_*H*_ for intermediate values of 1/*n* may be easily predicted by interpolating between the *s*_*H*_ values for CaGeO_3_ (with no interface) and Ca_2_GeO_4_ (maximum interface) rotation phases.

This steeper increase in *s*_*H*_ for rotation phases than high-symmetry phases with interface mole fraction (1/*n*) thus manifests as a large enhancement in κ between the child structure and parent, where the magnitude of this enhancement increases greatly with 1/*n*, reaching a maximum in the *n* = 1 structure. The key result of this analysis of the compliances is hence that this *n* = 1 structure is the best in the Ruddelsden-Popper series for facilitating uniaxial NTE.

### 3.2. Elastic compliances

Figure 3 showed how the eigenvalues of ***s*** vary with the CaGeO_3_:CaO interface fraction (1/*n*). In this section we take a step back and analyse how the individual components of the compliance matrix, *s*_*ij*_, vary with 1/*n*. In the second half of the section, we assess the quality of the two interpolations, (i) between CaGeO_3_ and CaO constituents and (ii) between CaGeO_3_ and CaGeO_3_:CaO interface constituents, to predict the compliance components of intermediate values of 1/*n*.

**Figure 4:**
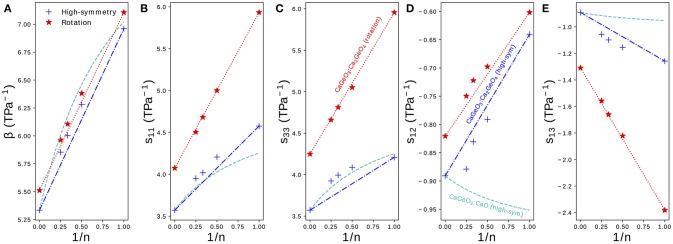
**(A)** The bulk compressibility, β and **(B**–**E)** components *s*_*ij*_ of the elastic compliance matrix for a tetragonal material plotted for high-symmetry and rotation phases in the Ca_*n*+1_Ge_*n*_O_3*n*+1_ series against the mole fraction of CaGeO_3_:CaO interface (1/*n*). Interpolations between values in the CaGeO_3_ (1/*n* = 0) and Ca_2_GeO_4_ (1/*n* = 1) structures are plotted for both high-symmetry and rotation phases (blue dot-dashed and red dotted lines, respectively). A third curve shows the interpolation between values in the $Pm\bar{3}m$ CaGeO_3_ phase and CaO rock salt structure computed as a function of CaO mole fraction 1/(*n* + 1) (cyan dashed).

Figure 4 shows the elastic compressibility, β, and components of the elastic compliance matrix, ***s***, computed for fully relaxed high-symmetry and rotation phases in the Ca_*n*+1_Ge_*n*_O_3*n*+1_ series. Since all phases are (pseudo-)tetragonal, only the four symmetrically distinct *s*_*ij*_ components identified in Equation (1) are plotted. The full ***s*** matrix, associated eigenvalues and eigenvectors and β may be found for all structures in Table S3.

The bulk elastic compressibility, β, increases linearly with 1/*n* but only very slight enhancement in compressibility is seen between the high-symmetry and rotation phases for a given *n*. Differences in β can therefore not be used to explain why uniaxial NTE is common in low *n* RP rotation phases but not in parent *I*4/*mmm* phases.

The normal compliance components, *s*_11_ and *s*_33_, also increase with 1/*n* but unlike in β there is a significant enhancement in the rotation phase compared to the high-symmetry parent, with both the magnitude and gradient with respect to 1/*n* greater in the rotation phase.

The sign of the off-diagonal compliance components, *s*_12_ and *s*_13_, that couple normal stresses to normal strains between axes, are negative for all compounds. This indicates that all materials have all positive Poisson ratios, ν_*ij*_, where ν_*ij*_ describes the normal strain of axis *j* in response to a strain of axis *i* ($\nu_{ij}=-\frac{\varepsilon_{j}}{\varepsilon_{i}}$). Most materials have ν_*ij*_ > 0, so these NTE RP phases are not auxetic (ν_*ij*_ < 0), even though auxetic materials have been linked with materials that exhibit anisotropic NTE (Wang et al., 2017).

Despite the negative sign, the behavior of *s*_13_ is similar to that of *s*_11_ and *s*_33_: compliance increases with 1/*n* and there is a large enhancement in both the magnitude and the gradient increase with 1/*n* in the NTE phase compared with the high-symmetry parent. *s*_12_, on the other hand, displays the opposite trend since the magnitude of coupling decreases with 1/*n* and going from the high-symmetry to rotation phases.

As in Figure 3, straight lines have been plotted on the subplots in Figure 4 interpolating between values for the CaGeO_3_ (1/*n* = 0) and Ca_2_GeO_4_ (1/*n* = 1) end members. A third dashed cyan line has been added to interpolate between the pure high-symmetry CaGeO_3_ and CaO rock salt constituent phases. Because the mole fraction of CaO is actually expressed as 1/(*n* + 1) (Equation 3) these lines appear curved when plotted against the 1/*n* x-axis.

The trend in β follows that that would be predicted by modeling the RP series as a laminate of CaGeO_3_ and CaO, suggesting that β is determined predominantly by the composition. Since bulk volume thermal expansion, α, is proportional to the bulk compressibility, β, this implies that the magnitude of α is heavily dependent on chemistry. This result echoes recent work showing that experimental measurements of many thermodynamic properties of RP structures may be predicted by interpolating between values of their chemical constituents (Glasser, 2017).

Whereas, β could be approximated well as a function of CaO content for RP phases, *s*_11_ of high-symmetry phases increases above that predicted by the cyan curve. This indicates that even in the high-symmetry phase, the CaGeO_3_ and CaO layers do not behave independently and are affected by the interface between them. The prediction for *s*_33_ based on the CaO content is quite good, which may be because *s*_33_ corresponds to deformations along the layering axis (with the *a* and *b* lattice parameters fixed) and therefore the different constituent layers are being stretched in series^1^. For both *s*_11_ and *s*_33_, the rotation phases follow a linear relationship with 1/*n* and therefore may be considered dependent upon the fraction of CaGeO_3_:CaO interface in the structure (red dotted line). However, in the high-symmetry phase the *n* > 1 values for both normal compliance components increase slightly beyond that predicted by interpolating between the extreme CaGeO_3_ and Ca_2_GeO_4_ values (blue dot-dashed line). This is surprising since it is not immediately obvious how the structure of higher *n* compounds is different to local regions of CaGeO_3_ and Ca_2_GeO_4_ and therefore what additional compliance mechanisms could operate.

For both *s*_12_ and *s*_13_, modeling the compliance according to the mole fraction of CaO is a poor approximation, so much so that this prediction actually gives the wrong sign of the change in *s*_12_ with 1/*n*.

### 3.3. Compliance enhancement mechanisms

**Figure 5:**
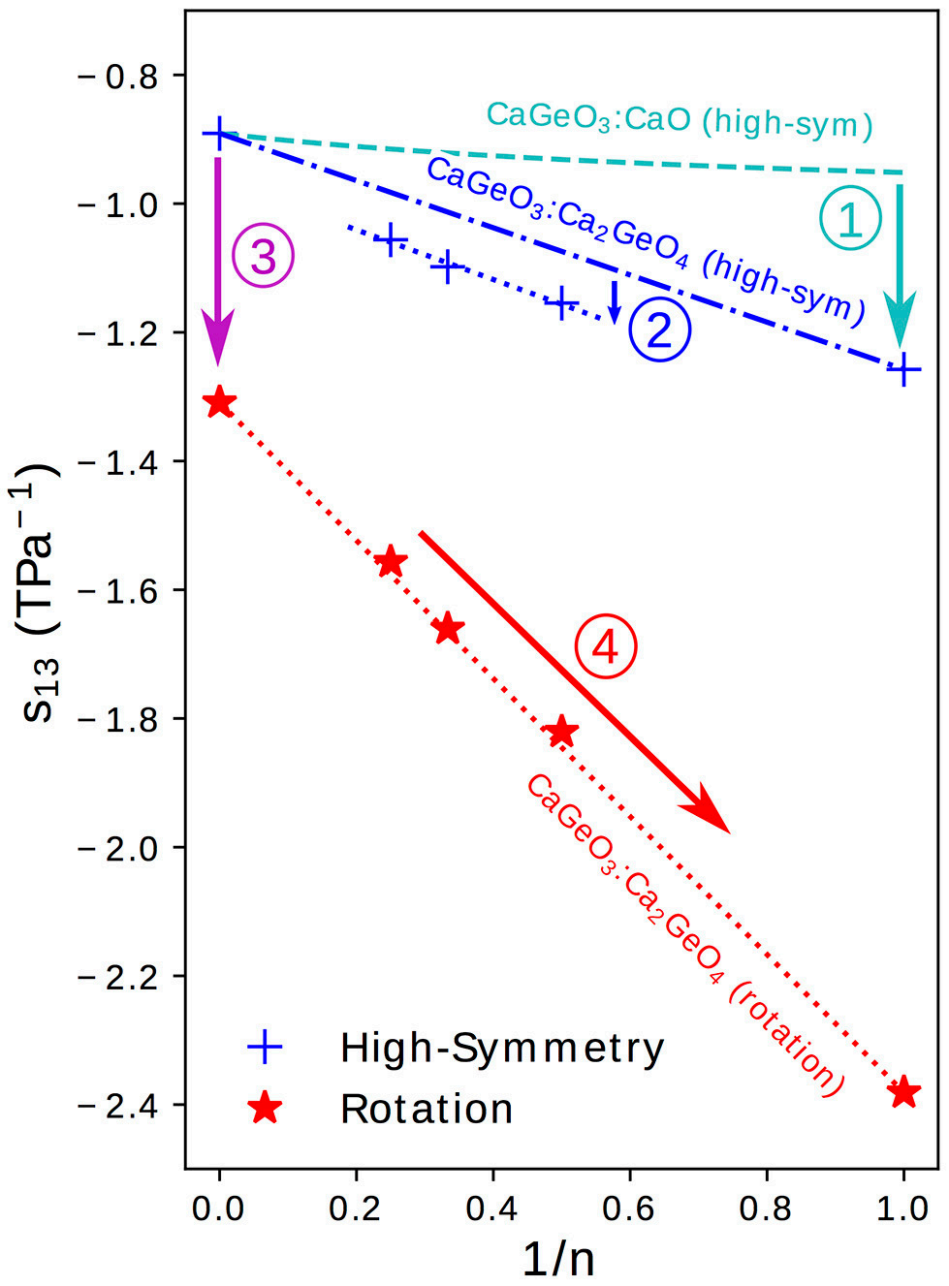
The *s*_13_ term of the elastic compliance matrix (corresponding to coupling between an in-plane axis, *a* or *b*, and the layering axis, *c*) for a tetragonal material plotted for high-symmetry and rotation phases in the Ca_*n*+1_Ge_*n*_O_3*n*+1_ series against the mole fraction of CaGeO_3_:CaO interface (1/*n*). Numbered arrows show the enhancement in compliance between different regimes represented by lines.

In section 3.2 we showed that certain elastic properties, such as the bulk compressibility, β, are insensitive to small changes in crystal symmetry and may be accurately predicted by interpolating between the value of β in CaGeO_3_ and CaO end member structures based on the mole fraction of CaO. However, components of the anisotropic compliance matrix, *s*_*ij*_, typically differ in magnitude between high and low symmetry phases and are generally more compliant than a CaGeO_3_:CaO interpolation predicts. Figure 5 shows the same plot as in Figure 4E (*s*_13_ vs. 1/*n*) but with annotations decomposing the *s*_13_ behavior of different structures into regimes of increasingly enhanced compliance. By separating the compliance regimes in this way, in this section we discuss the atomic displacements allowed in each regime by the phase symmetry and thus propose atomic mechanisms that may explain these enhancements in the *s*_13_ axis coupling parameter. In many cases (although not discussed here) this analysis may be used to explain the different regimes of the *s*_11_, *s*_33_, and *s*_12_ components in Figure 4.

**Figure 6:**
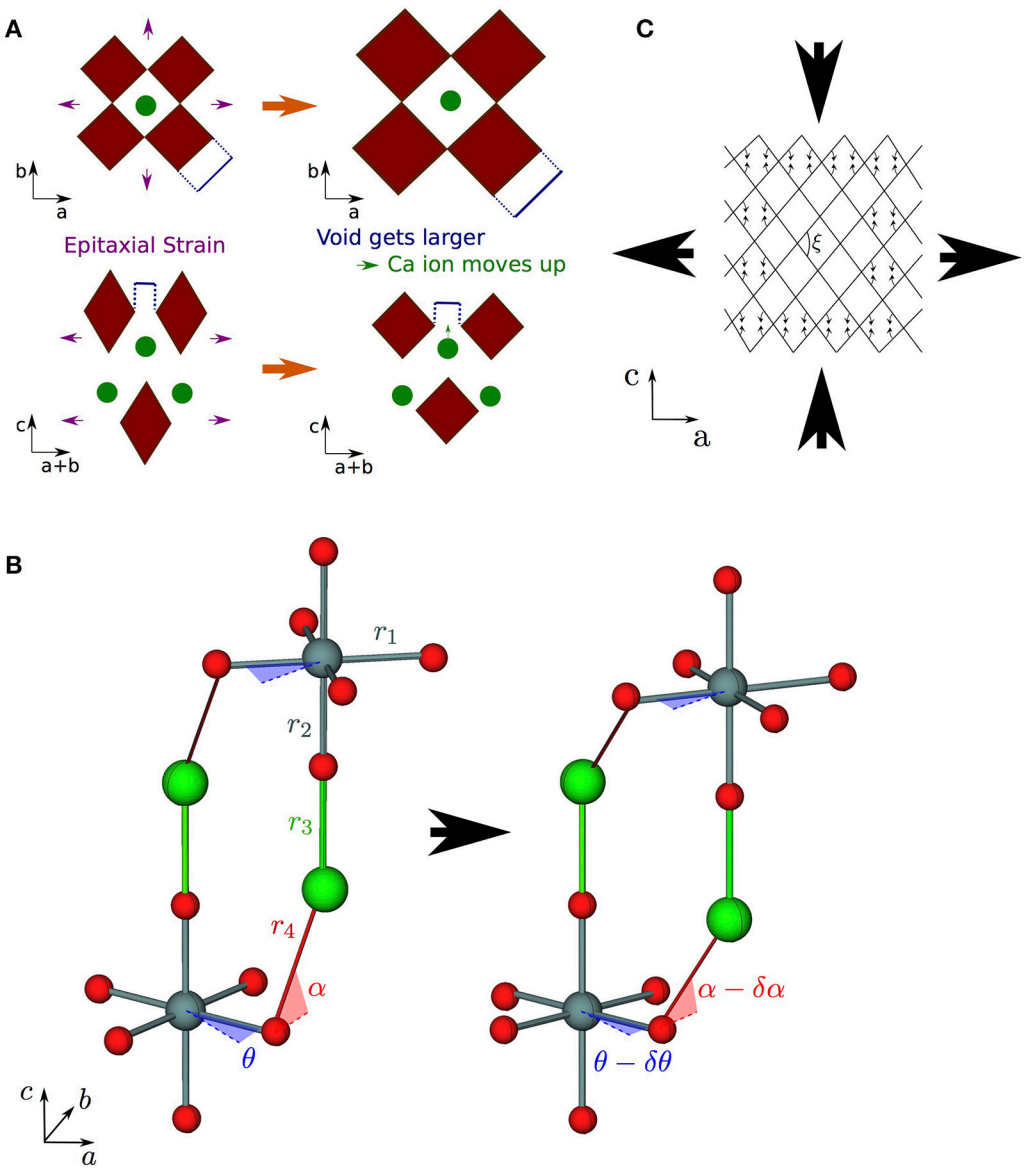
Strain coupling mechanisms that make structures particularly compliant to cooperative strains. Since these mechanisms couple the *a* (and/or *b*) and *c* axes, they are typified by large *s*_13_ off-diagonal components of the compliance matrix. **(A)** Mechanism for increased compliance in *I*4/*mmm* RP structures where the rumpling between the interfacial A and apical O ions means that A is free to displace independently of O in response to in-plane strains; **(B)** corkscrew mechanism in RP rotation phases—the structure is able to couple in-plane tensile strains to compressive strains along *c* via internal displacements assuming all nearest neighbor B–O bonds and the two shortest A–O bonds do not deform (shown as rigid struts labeled *r*_1_–*r*_4_) by changing the angle of in-plane and out-of-plane hinges (described by the angles θ and α as shown); **(C)** wine rack structure where tensile strains along *a* may couple to compressive strains along *c* in a system of rigid struts in a trellis structure by only changing the angle ξ.

Taking the value of *s*_13_ that would be predicted by interpolating between values in the CaGeO_3_ and CaO constituent structures as a base (the dashed cyan curve in Figure 5), arrow ① represents an increase in the coupling between in-plane (*a* and *b*) axes and the layering axis (*c*) in the *n* = 1 Ca_2_GeO_4_ *I*4/*mmm* phase. In a pure cubic ABO_3_ perovskite, the A cations by symmetry have the same *z* position as the apical O anions. However, the inclusion of the AO layer in high-symmetry RP phases causes symmetry breaking along *c* at the ABO_3_:AO interface such that the apical O and interfacial A ions are no longer restricted to the same *z* coordinate, leading to a so-called “rumpling” of the AO layer. We propose that this rumpling facilitates a mechanism for enhanced *s*_13_ coupling illustrated in Figure 6A: As the in-plane, *ab*, axes are strained, the interstitial void between BO_6_ octahedra below the interfacial A cation changes in size, but the rumpling adds a degree of freedom to the *z* coordinate of the A cation which may thus move further into/out of the void in response to the in-plane strain. This thus couples the in-plane, *ab*, axes to internal displacements along the layering axis *c* and therefore to the layering axis itself.

It was commented in the preceding section that the enhancement in compliance from the interpolation between CaGeO_3_ and Ca_2_GeO_4_ values to *n* > 1 high-symmetry RP structures (shown by arrow ② in Figure 5) is surprising since all structures consist of a CaGeO_3_:CaO interface (with rumpling of the CaO *z* positions) and blocks of CaGeO_3_ (that one might expect to behave as bulk cubic CaGeO_3_). Close inspection of the *n* = 1 and *n* = 2 *I*4/*mmm* phases in Figure 1 allows us to see that in A_2_BO_4_ structures, the length of all apical B–O bonds are equal due to the mirror symmetry plane lying in each BO_6_ layer. Similarly the angle between epitaxial B–O and apical B–O bonds must be 90° by the same reasoning. However, in A_3_B_2_O_7_ *I*4/*mmm* phases, this restriction that the perovskite blocks must contain a mirror plane at the centre only means that the two outer apical B–O bond lengths and BO_6_ internal angles must be equal and the apical B–O bond lengths between the two inner BO_6_ layers must be equal. There is no restriction by symmetry that all apical B–O bond lengths must be equal or in fact that all BO_6_ internal angles must be 90°. These weaker restrictions create internal degrees of structural freedom that may facilitate greater compliance, since there is greater freedom for the atoms to move in response to external strains. In structures relaxed using DFT, we find that there are slight differences in these two bondlengths: 1.87 and 1.90 Å for the outer and inner apical B–O bonds, respectively, and the angle between outer apical B–O and epitaxial B–O bonds is 91.2°. This same argument may be applied to all *n* > 1 *I*4/*mmm* phases. However, in the special case of the *n* = ∞ series end member, there is no AO layer to break any symmetry along *c* and thus all B–O bond lengths are equal and all O–B–O angles are 90°. That this difference between *s*_13_ of *n* = 2, 3, 4 high-symmetry phases and the CaGeO_3_:Ca_2_GeO_4_ interpolation is not also seen in the significantly more compliant rotation phases may be because in these rotation phases, additional compliance mechanisms operate that dwarf the effect described by arrow ②.

Arrow ③ in Figure 5 represents the increase in coupling between the *a*/*b* and *c* axes from the cubic CaGeO_3_ phase to an *I*4/*mcm* phase with anti-phase octahedral rotation about the *c* axis. From a structural symmetry point of view, this may again come as a surprise since the *I*4/*mcm* phase only has an internal degree of freedom in the *ab* plane and not along the *c* axis that this plane couples to. Therefore, one might expect large changes in in-plane strain in response to biaxial stress from rigid BO_6_ octahedra rotating, but not also large changes in *c*. However, DFT studies on LaAlO_3_ (Hatt and Spaldin, 2010) and LaNiO_3_ (Weber et al., 2016) show that while the application of compressive biaxial strain to *I*4/*mcm* phases causes the rotation angle to increase, it also leads to a large tetragonal distortion of the BO_6_ units with compression of the epitaxial B–O bonds and extension of the apical B–O bonds.

Finally, arrow ④ in Figure 5 represents the increase in *s*_13_ coupling between rotation phases of CaGeO_3_ perovskite and RP phases with a frozen octahedral rotation. In these phases, the rumpling of the interfacial AO layer identified in the high-symmetry structure is still present. However, whereas in the high-symmetry structure, in-plane strains necessarily involved deformations of stiff epitaxial B–O bonds, in this lower-symmetry phase, the frozen in-plane octahedral rotation adds an internal degree of freedom in the in-plane epitaxial O positions. There are thus internal degrees of freedom in both in-plane and layering axes in RP rotation phases. In our previous paper (Ablitt et al., 2017) we proposed a “corkscrew” mechanism to explain high *s*_13_ coupling in RP1 rotation phases but not in *I*4/*mcm* ABO_3_ phases. This coupling mechanism in theory allows *a* and *c* to deform cooperatively without extending the four most stiff cation-anion bonds identified in the *I*4_1_/*acd* Ca_2_GeO_4_ structure, by only changing two bond angles, labeled θ and α in Figure 6B. We call this mechanism corkscrew since an in-plane rotation leads to an extension along the rotation axis: Figure 6B shows how the in-plane rotation angle θ may decrease in response to a biaxial expansion, such that this in turn pulls the stiff O-A bond (shown as a rigid rod), decreasing the angle α and thus increasing the rumpling of the rock salt layer and forcing contraction along the *c*-axis.

The net result of these interfacial strain coupling mechanisms in RP phases, yielding an enhanced off-diagonal *s*_13_ compliance term, is rather reminiscent of the “wine-rack” mechanism such as that which operates in methanol monohydrate (Fortes et al., 2011). In the wine-rack trellis structure, shown schematically in Figure 6C with rigid struts but flexible hinges, *a* couples strongly to *c*, mediated by the hinge angle, ξ, in the *ac* plane. Following the method used to analyse the wine-rack (Grima and Evans, 2000) and other idealized geometries (Smith et al., 2000, Grima et al., 2012), we were able to derive the mechanical properties that our pure corkscrew mechanism would exhibit under the conditions that the four *stiff* bonds shown in Figure 6B indeed remained rigid and all resistance to strain came from a harmonic potential in the θ and α-hinges (Ablitt et al., 2018). Under these restrictions, a corkscrew model would have an *s*_13_ compliance parameter as a function of RP layer thickness, *n*, given by

(4) $$\begin{array}{l} s_{13}=-\frac{\text{f}\left(\theta ,\alpha \right)r_{1}^{3}}{\left[nk_{\theta }+k_{\alpha }{\left(\frac{d\alpha }{d\theta }\right)}^{2}\right]}, \end{array}$$

where *k*_θ_ and *k*_α_ represent the harmonic stiffness of the θ and α-hinges, respectively, and f(θ, α) is an expression of trigonometric functions of θ and α

(5) $$\begin{array}{l} \text{f}\left(\theta ,\alpha \right)=\frac{\sin\left(2\theta \right)\left[\sin\left(\theta \right)+\cos\left(\theta \right)\right]}{4\tan\left(\alpha \right)}. \end{array}$$

Under the constraints of this model, α is explicitly dependent on θ by the equation

(6) $$\begin{array}{l} r_{4}\cos\left(\alpha \right)=r_{1}\left[\cos\left(\theta \right)-\sin\left(\theta \right)\right], \end{array}$$

and therefore given the bond lengths *r*_1_-*r*_4_ (as defined in Figure 6B), the value of θ fully determines the structure.

In the limit that *k*_α_ >> *k*_θ_, the AO interface is stiffer than the ABO_3_ perovskite blocks and *s*_13_ loses dependence on *n*. However, in the opposing limit that *k*_θ_ >> *k*_α_ and changing the in-plane rotation angle is the main obstacle to strain, *s*_13_∝1/*n*. The real system is closer to the *k*_θ_ >> *k*_α_ limit as we observe linear behavior of *s*_13_ with 1/*n*. Although this model has been derived assuming that all equatorial B–O bond lengths (*r*_1_) and all apical B–O bond lengths (*r*_2_) are equal, the bond lengths *r*_2_ and *r*_3_ do not feature in Equation (4) and *r*_1_ and θ only need refer to the in-plane bond lengths and rotation angle in the outer layer of the perovskite block. Therefore, this mechanism is compatible with a distribution of possible bond lengths and rotation angles in different layers of a perovskite block if *n* > 1.

We accept the limitations of such simple models but developing the study of how symmetry-allowed local distortions can give rise to new compliance mechanisms in different crystallographic phases, such as those identified using arrows ①–④ in Figure 5, may allow for the prediction of phases with high cross compliances by symmetry alone, before needing to explicitly compute the elastic constants. Calculations of elastic constants are, in turn, frequently less expensive than performing lattice dynamics calculations across the full Brillouin zone to compute the thermodynamic driving force for anisotropic thermal expansion, Φ.

Therefore, looking for other materials with such high cross compliances, using symmetry analysis as a guide to narrow the pool of structures, may prove a more general method for searching for novel NTE materials. Indeed, by considering this analysis and our thermodynamic criteria requiring a proximity to a competing phase transition to provide Φ, we have already been able to identify (Ablitt et al., 2017) the layered double perovskites Sr_2_MgWO_6_ (Achary et al., 2006) and (110)-cut perovskite LaTaO_4_ (Cordrey et al., 2015) which fall within this general paradigm. Substantial research opportunities exist in this area to more fully explore the NTE behavior of these classes of materials.

### 3.4. Dynamic driving force for NTE

**Figure 7:**
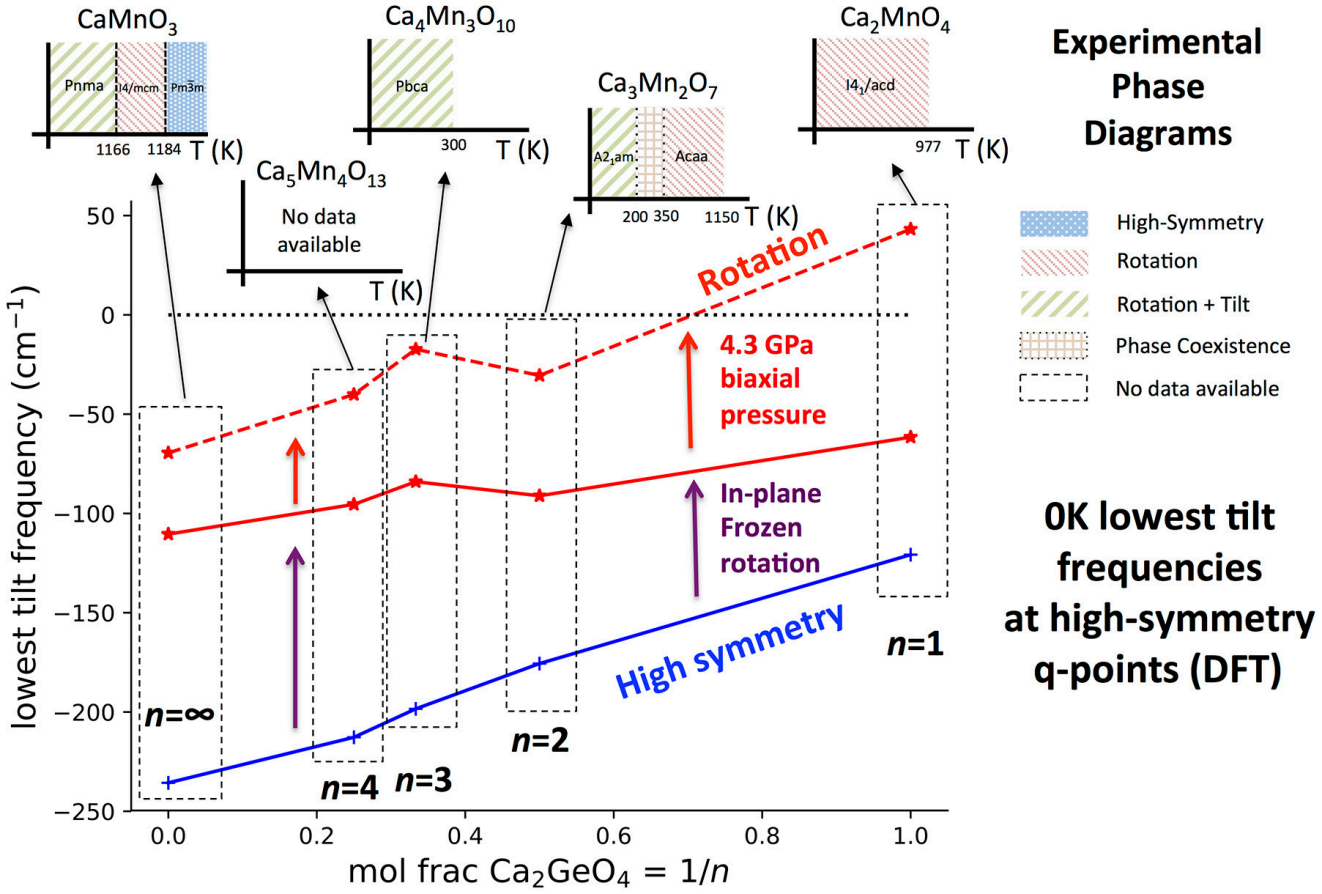
The lowest frequency harmonic phonon mode with octahedral tilt character found in DFT simulations at high-symmetry q-points in the Ca_*n*+1_Ge_*n*_O_3*n*+1_ series against interface fraction, 1/*n*. Tilt frequencies have been plotted in the high-symmetry and rotation phases with 0 GPa external pressure, and also for the rotation phase with a 4.3 GPa biaxial pressure applied to give better agreement of the Ca_*n*+1_Ge_*n*_O_3*n*+1_ structure to Ca_*n*+1_Mn_*n*_O_3*n*+1_. DFT only simulates the system at 0 K whereas phonon frequencies may harden with increased temperature. This means that many octahedral tilts are predicted with imaginary frequencies (shown as negative) even if the mode has a real frequency and the structure is stable at some higher temperature. To give an idea of how the equilibrium phase changes with temperature, the inset graphs plot the experimentally observed phase diagram for each *n* in the Ca_*n*+1_Mn_*n*_O_3*n*+1_ system. The data to make these illustrations was taken from: Ca_2_MnO_4_ (Takahashi and Kamegashira, 1993); Ca_3_Mn_2_O_7_ (Senn et al., 2015); Ca_4_Mn_3_O_10_ (Battle et al., 1998); CaMnO_3_ (Taguchi et al., 1989).

So far we have not addressed the thermodynamic driving force for thermal expansion Φ(*T*), and it is rather more computationally expensive to calculate than κ as it requires the full phonon density of states to be computed for different strained structures within the QHA. Furthermore, this procedure is only possible if all phonons within the NTE structure simulated using DFT have real frequencies—suggesting that the phase must be stable at 0 K. For *n* > 1 structures in the Ca_*n*+1_Ge_*n*_O_3*n*+1_ series, this latter condition is not met, and therefore full computation of Φ(*T*) as we performed previously (Ablitt et al., 2017) would not be possible. However, we also previously identified that the most important modes driving NTE are octahedral tilts about an in-plane axis. Thus in Figure 7 we have plotted the frequency of the lowest frequency tilt mode to occur at a high-symmetry q-point in high-symmetry phases and phases with a frozen in-plane rotation. We note that these tilts are not the same as the vibrations of ions along the *z* direction predicted using a QHA-inspired method to cause NTE in a *A*2_1_*am* Ca_3_Ti_2_O_7_ phase after 30 GPa hydrostatic pressure has been applied and in which these octahedral tilts are already frozen (Huang et al., 2016). As in previous studies (Senn et al., 2016, Ablitt et al., 2017) the Ca_*n*+1_Ge_*n*_O_3*n*+1_ series is being treated as an analog to Ca_*n*+1_Mn_*n*_O_3*n*+1_ for comparison against experimental data to avoid expensive magnetic calculations since Ge^4+^ and Mn^4+^ are known to have equal ionic radii (Shannon, 1976). The authors have previously shown that this substitution has little bearing on the properties (phonon frequencies, elastic constants) relevant to modeling thermal expansion within the QHA.

It can be seen from Figure 7 that in all phases, the frequency of the softest tilt mode stiffens with increased Ca_2_GeO_4_ content, indicating that the inclusion of the CaGeO_3_:CaO interface reduces the propensity of octahedra to tilt. The tilt mode is unstable (has an imaginary frequency) in all high-symmetry parent phases, which is unsurprising since these phases are not observed experimentally at low temperatures at any *n* for the Ca_*n*+1_Mn_*n*_O_3*n*+1_ series. The tilt stiffens between the parent and child phases for all *n*, showing that the dynamic tilt couples competitively with the frozen in-plane rotation. In both the high- and low-symmetry phases, the (imaginary) octahedral tilt frequency varies approximately linearly with Ca_2_GeO_4_ mole fraction, although the stiffening effect going between the high- and low- symmetry phases becomes greater at higher *n* (lower Ca_2_GeO_4_ fraction). The CaO rock salt layer also stiffens octahedral rotations and thus the angle of the frozen rotation increases with *n* in rotation phases from 10.55° in *I*4_1_/*acd* Ca_2_GeO_4_ (*n* = 1) up to the limit of 12.7° in *I*4/*mcm* CaGeO_3_ (*n* = ∞; see Figure S2). Hence, the greater amplitude of frozen rotation observed in CaGeO_3_ means that the competitive coupling between the frozen rotation and dynamic tilt is greatest for this largest *n* member, and leads to further hardening of the dynamic tilt frequency.

In the 0 GPa relaxed Ca_*n*+1_Ge_*n*_O_3*n*+1_ structures, the tilt is still unstable for all *n*. However, it was found previously (Ablitt et al., 2017) that a discrepancy arises between the Ca_2_GeO_4_ and Ca_2_MnO_4_ in-plane lattice parameters in the *I*4_1_/*acd* phase—due to in-plane magneto-strictive coupling—but that applying a biaxial 4.3 GPa pressure corrects for this small difference, yielding very close agreement in the frequencies of the softest phonon modes. After applying this biaxial pressure, the *n* = 1 *I*4_1_/*acd* Ca_2_GeO_4_ rotation phase has all real mode frequencies, but the softest tilt in all structures with *n* > 1 is still unstable. This agrees with experimental observation of the low temperature stable phases, shown as inset figures for each composition on Figure 7.

Ca_3_Mn_2_O_7_ is found at low temperature in the improper ferroelectric *A*2_1_*am* phase, but undergoes a wide temperature region of phase coexistence with the uniaxial NTE *Acaa* phase between 150 and 280 K on cooling and 300 and 360 K on heating (Senn et al., 2015). The strong first order nature of the phase transition arises because frozen octahedral rotations in the *A*2_1_*am* and *Acaa* phases have opposite sense (in-phase vs. out-of-phase about *c* within each perovskite layer—see Figure A1 in Appendix 2) but the approximate transformation temperature gives an indication of the temperature at which octahedral tilts in a rotation phase develop real frequencies. Furthermore, the *n* = ∞ perovskite, CaMnO_3_, transforms around 1166 K from a *Pnma* ground state with frozen octahedral tilts, to an *I*4/*mcm* phase with only an out-of-phase octahedral rotation—the *n* = ∞ analog of the *n* = 2 *Acaa*—remaining in this phase for only a brief temperature window before transforming again to the cubic parent structure at 1,184 K (Taguchi et al., 1989). For *n* = 3, the reported symmetry for Ca_4_Mn_3_O_10_ from 5 K up until room temperature is *Pbca* (Battle et al., 1998), in which a static rotation and tilt of the octahedra are present, and, although to the best of our knowledge a transformation to a higher-symmetry phase with only a frozen octahedral rotation has not yet been reported^2^, interpolating the experimental observations in Figure 7 predicts a transformation temperature in the 250–1,100 K window. The magnitude of the imaginary tilt frequency computed in Figure 7 may therefore by interpreted as a crude indicator of the stability of the structure with condensed rotation and tilt and thus of the temperature required to transform to the higher-symmetry rotation phase.

As well as having optimal elastic anisotropy to facilitate uniaxial NTE, the *n* = 1 Ca_2_MnO_4_ *I*4_1_/*acd* phase has soft tilt modes at low temperatures providing a thermodynamic driving force for cooperative in-plane positive and out-of-plane NTE. This feature is not unique to the *n* = 1 structure however, since at some higher temperature all Ca_*n*+1_Mn_*n*_O_3*n*+1_ compounds should transform to a phase in which the tilt frequencies are real and soft, at least over some temperature range. Furthermore, we have demonstrated previously in the Ca_3−*x*_Sr_*x*_Mn_2_O_7_ system, that for a given layer thickness *n*, chemical substitution (changing *x*) may be used to alter the Goldschmidt tolerance factor and thus the frequencies of these octahedral tilts, switching between positive and negative uniaxial thermal expansion (Senn et al., 2016). Therefore, Figure 7 shows that the RP structure, through the layer thickness *n*, determines a ballpark value for the 0 K tilt frequency in the uniaxial NTE phase, and thus a ballpark temperature window in which the NTE phase will be stable. Hence, given this structural constraint we may use chemical control to optimize the proximity of the structure to the phase transition and so enhance the NTE. In this respect, the *n* = 1 family member, since one may presume that it may be tuned chemically to arbitrary proximity to the competing phase transition, is the most promising candidate for exhibiting the largest NTE on account of the anisotropy with respect to its most and least compliant directions which we have shown is maximized for this system.

## 4. Conclusions

We have shown that the elastic anisotropy ratio, κ, found previously to be an essential ingredient for uniaxial NTE, increases linearly in the RP Ca_*n*+1_Ge_*n*_O_3*n*+1_ series (*n* = 1, 2, 3, 4 …∞) with the CaGeO_3_:CaO content (expressed by the ratio 1/*n*), reaching a maximum in the structure with maximal interface (*n* = 1). By decomposing the components of the elastic compliance matrix for high-symmetry and NTE phases (with a frozen octahedral rotation about the layering axis) into different regimes that show similar trends with 1/*n*, we have been able to link these regimes with internal degrees of freedom in the structure that allow atomic mechanisms to operate that couple to cell strains. The most important of these is the “corkscrew” mechanism that operates locally at the CaGeO_3_:CaO interface in phases with a frozen octahedral rotation about the layering axis and therefore explains the trend that anisotropic compliance correlates with the fraction of interface in these phases. This local atomic compliance mechanism is analogous in certain ways to the wine-rack mechanism that operates in many much softer framework materials. The compliance matrices can be rapidly calculated by DFT methods and diagonalized to assess them for cross coupling terms that promote pronounced uni or biaxial NTE. This makes them suitable descriptors for high throughput computational searching for novel NTE materials, especially when symmetry constraints may be employed to narrow the space of candidate phases.

We further investigated the trend in frequency of octahedral tilts with RP layer thickness and found that the 0 K tilt frequencies in NTE or analogous structures become softer with increasing *n*. This implies that a window of stability of the NTE phase with soft active tilt modes exists at increasingly higher temperatures with higher *n*. We had previously shown that the thermodynamic driver for NTE for a given *n* might be tuned with chemical substitution, and we now show that the anisotropic compliance necessary for NTE in these systems is maximized for a high fraction of CaGeO_3_:CaO interface layers in the structure. On the basis of this analysis, we thus predict that the *n* = 1 systems, such as Ca_2_MnO_4_, will be the RP systems in which the maximum NTE can be achieved via chemical substitution.

## Data availability statement

Data underlying this article can be accessed on figshare at DOI:10.6084/m9.figshare.6729287, and used under the Creative Commons Attribution licence.

## Author contributions

CA performed the calculations and data analysis. All authors contributed to the design of the study and the analysis and interpretation of the results. The paper was drafted by CA and MS, and all authors contributed to its development into final form.

### Conflict of interest statement

The authors declare that the research was conducted in the absence of any commercial or financial relationships that could be construed as a potential conflict of interest.

## Footnotes

^1^However, we note that under these conditions one should use a Voigt average to interpolate *s*_33_, whereas by drawing a straight line of compliance vs mole fraction we have actually performed a Reuss interpolation. In Figure S1 in the SI we show that a Voigt fit against CaO mole fraction is actually less close.

^2^Although we do note that the high-symmetry *I*4/*mmm* parent structure has been stabilized at room temperature from solid state synthesis at high temperatures and pressures (Yu et al., 2001).
