# Supplementary material for: Control of Uniaxial Negative Thermal Expansion in Layered Perovskites by Tuning Layer Thickness
Source: Front Chem. 2018 Oct 18;6:455. doi: 10.3389/fchem.2018.00455 (PMC6201142; doi:10.3389/fchem.2018.00455)
Supplement: Supplementary file 1 [file Data_Sheet_1.PDF]

Supplementary Material: Control of Uniaxial Negative Thermal Expansion in Layered Perovskites by Tuning Layer Thickness

1 MECHANICS OF LAYERED COMPOSITES

The equations in this section relate to Figure S1.

The Reuss interpolation of a compliance s for a $Ca_{1+n}Ge_nO_{3n+1}$ structure with layer thickness n in terms of the values of s in the $CaGeO_3$ and CaO structures ($s\left(CaGeO_3\right)$ and $s\left(CaO\right)$ respectively is

$$s\left(n\right)=\left(\frac{1}{1+n}\right)s\left(\mathrm{CaO}\right)+\left(1-\frac{1}{1+n}\right)s\left(\mathrm{CaGeO_3}\right), \tag{S1}$$

meanwhile the Voigt interpolation is

$$s\left(n\right)^{-1}=\left(\frac{1}{1+n}\right)s\left(\mathrm{CaO}\right)^{-1}+\left(1-\frac{1}{1+n}\right)s\left(\mathrm{CaGeO_3}\right)^{-1}. \tag{S2}$$

The Reuss interpolation provides an upper limit on the bulk compressibility of a composite whereas the Voigt interpolation provides a lower limit within composite mechanics theory. The Reuss interpolation assumes equal stresses between different layers and therefore is most appropriate for modelling laminates loaded along the layering axis. The Voigt interpolation on the other hand assumes strains are equal and thus is most appropriate for loading along directions in the layer plane.

2 SUPPLEMENTARY TABLES AND FIGURES

Ca	3|2.0|9|10|12|30N:40N:31N:32N(qc=6)
Ge	3|1.8|28|32|38|40N:41N:32N1.5(qc=5,q2=10)
O	1|1.2|23|26|31|20N:21L(qc=9)

Table S1. Pseudopotential strings used in all DFT calculations, generated on-the-fly within CASTEP version 16.0

n	Chemistry	Phase	a (Å)	b (Å)	c (Å)	ΔE (meV/at)
1	Ca_2GeO_4	$I4/mmm$	3.749	3.749	11.646	0.0
1	Ca_2GeO_4	$Acam$	5.234	5.234	11.945	-9.1
1	Ca_2GeO_4	$I4_1/acd$	5.234	5.234	23.889	-9.1
2	$Ca_3Ge_2O_7$	$I4/mmm$	3.754	3.754	19.074	0.0
2	$Ca_3Ge_2O_7$	$Acaa$	5.229	5.230	19.612	-22.0
3	$Ca_4Ge_3O_{10}$	$I4/mmm$	3.753	3.753	26.559	0.0
3	$Ca_4Ge_3O_{10}$	$Acam$	5.227	5.227	27.282	-29.6
3	$Ca_4Ge_3O_{10}$	$I4_1/acd$	5.227	5.227	54.564	-29.6
4	$Ca_5Ge_4O_{13}$	$I4/mmm$	3.752	3.752	34.054	0.0
4	$Ca_5Ge_4O_{13}$	$Acaa$	5.226	5.226	34.954	-34.0
∞	$CaGeO_3$	$Pm3m$	3.748	3.748	3.748	0.0
∞	$CaGeO_3$	$I4/mcm$	5.221	5.221	7.672	-49.2

Table S2. Table of all relaxed cell parameters from DFT calculations with no external pressure applied. Energies are given compared to the parent phase.

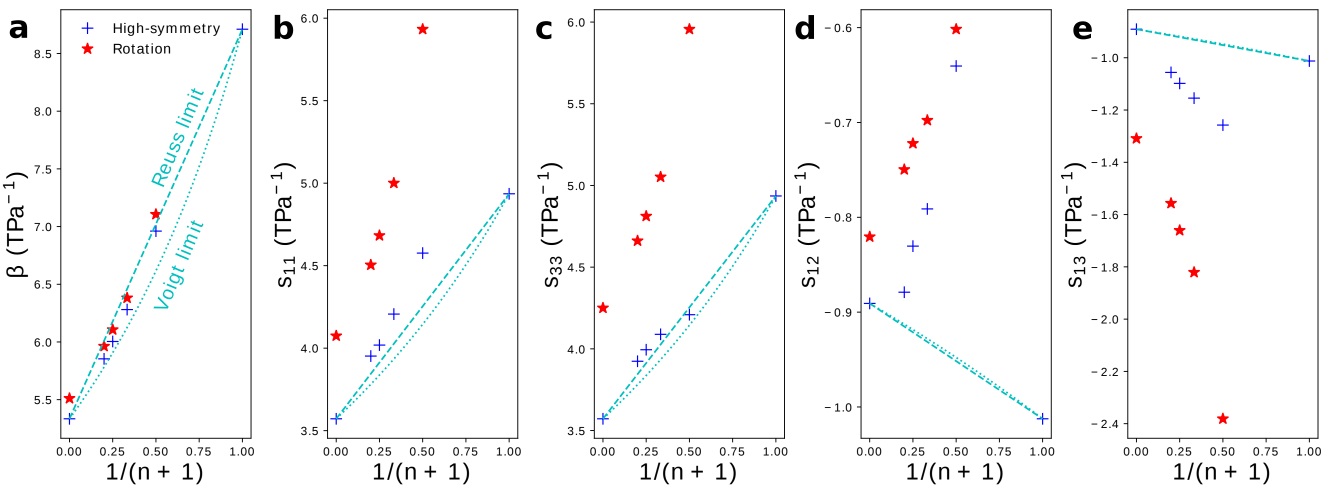

Figure S1. a) Lowest, s_L and highest, s_H, eigenvalues to the compliance matrix, $\boldsymbol{s}$, and b) the anisotropy ratio κ plotted for high-symmetry and rotation phases in the $Ca_{1+n}Ge_nO_{3n+1}$ series against the mole fraction of CaO $(1/(n+1))$. Reuss (Equation S1) and Voigt (Equation S2) interpolations between values in the $CaGeO_3$ $(1/(n+1) = 0)$ and CaO $(1/(n+1) = 1)$ structures are plotted for each variable s (dashed and dotted cyan curves respectively).

n	Chemistry	Phase	β	$\boldsymbol{s}$	s_L	$\hat{s}_L$	s_M	$\hat{s}_M$	s_H	$\hat{s}_H$
1	Ca_2GeO_4	$I4/mmm$	6.69	$\begin{bmatrix} 4.58 & -0.64 & -1.26 \\ -0.64 & 4.58 & -1.26 \\ -1.26 & -1.26 & 4.21 \end{bmatrix}$	2.29	$\begin{pmatrix} 0.52 \\ 0.52 \\ 0.68 \end{pmatrix}$	5.22	$\begin{pmatrix} 0.71 \\ -0.71 \\ 0.00 \end{pmatrix}$	5.86	$\begin{pmatrix} -0.48 \\ -0.48 \\ 0.73 \end{pmatrix}$
		$I4_1/acd$	7.11	$\begin{bmatrix} 5.93 & -0.60 & -2.38 \\ -0.60 & 5.93 & -2.38 \\ -2.38 & -2.38 & 5.96 \end{bmatrix}$	2.26	$\begin{pmatrix} 0.52 \\ 0.52 \\ 0.67 \end{pmatrix}$	6.53	$\begin{pmatrix} 0.71 \\ -0.71 \\ 0.00 \end{pmatrix}$	9.03	$\begin{pmatrix} -0.48 \\ -0.48 \\ 0.74 \end{pmatrix}$
2	$Ca_3Ge_2O_7$	$I4/mmm$	6.28	$\begin{bmatrix} 4.21 & -0.79 & -1.15 \\ -0.79 & 4.21 & -1.15 \\ -1.15 & -1.15 & 4.09 \end{bmatrix}$	2.09	$\begin{pmatrix} 0.55 \\ 0.55 \\ 0.63 \end{pmatrix}$	5.00	$\begin{pmatrix} 0.71 \\ -0.71 \\ 0.00 \end{pmatrix}$	5.42	$\begin{pmatrix} -0.45 \\ -0.45 \\ 0.78 \end{pmatrix}$
		$Acaa$	6.38	$\begin{bmatrix} 5.00 & -0.70 & -1.82 \\ -0.70 & 5.00 & -1.82 \\ -1.82 & -1.82 & 5.05 \end{bmatrix}$	2.07	$\begin{pmatrix} 0.53 \\ 0.53 \\ 0.65 \end{pmatrix}$	5.70	$\begin{pmatrix} 0.71 \\ -0.71 \\ -0.00 \end{pmatrix}$	7.28	$\begin{pmatrix} -0.46 \\ -0.46 \\ 0.76 \end{pmatrix}$
3	$Ca_4Ge_3O_{10}$	$I4/mmm$	6.00	$\begin{bmatrix} 4.02 & -0.83 & -1.10 \\ -0.83 & 4.02 & -1.10 \\ -1.10 & -1.10 & 3.99 \end{bmatrix}$	1.99	$\begin{pmatrix} 0.56 \\ 0.56 \\ 0.61 \end{pmatrix}$	4.85	$\begin{pmatrix} 0.71 \\ -0.71 \\ 0.00 \end{pmatrix}$	5.20	$\begin{pmatrix} -0.43 \\ -0.43 \\ 0.79 \end{pmatrix}$
		$Acam$	6.10	$\begin{bmatrix} 4.68 & -0.72 & -1.66 \\ -0.72 & 4.68 & -1.66 \\ -1.66 & -1.66 & 4.81 \end{bmatrix}$	2.00	$\begin{pmatrix} 0.54 \\ 0.54 \\ 0.64 \end{pmatrix}$	5.40	$\begin{pmatrix} 0.71 \\ -0.71 \\ 0.00 \end{pmatrix}$	6.77	$\begin{pmatrix} -0.45 \\ -0.45 \\ 0.77 \end{pmatrix}$
4	$Ca_5Ge_4O_{13}$	$I4/mmm$	5.85	$\begin{bmatrix} 3.95 & -0.88 & -1.06 \\ -0.88 & 3.95 & -1.06 \\ -1.06 & -1.06 & 3.92 \end{bmatrix}$	1.95	$\begin{pmatrix} 0.56 \\ 0.56 \\ 0.60 \end{pmatrix}$	4.83	$\begin{pmatrix} 0.71 \\ -0.71 \\ -0.00 \end{pmatrix}$	5.05	$\begin{pmatrix} -0.43 \\ -0.43 \\ 0.80 \end{pmatrix}$
		$Acaa$	5.96	$\begin{bmatrix} 4.50 & -0.75 & -1.56 \\ -0.75 & 4.50 & -1.56 \\ -1.56 & -1.56 & 4.66 \end{bmatrix}$	1.96	$\begin{pmatrix} 0.55 \\ 0.55 \\ 0.63 \end{pmatrix}$	5.25	$\begin{pmatrix} 0.71 \\ -0.71 \\ 0.00 \end{pmatrix}$	6.46	$\begin{pmatrix} -0.45 \\ -0.45 \\ 0.78 \end{pmatrix}$
∞	$CaGeO_3$	$Pm\bar{3}m$	5.33	$\begin{bmatrix} 3.57 & -0.89 & -0.89 \\ -0.89 & 3.57 & -0.89 \\ -0.89 & -0.89 & 3.57 \end{bmatrix}$	1.79	$\begin{pmatrix} 0.58 \\ 0.58 \\ 0.58 \end{pmatrix}$	4.46	$\begin{pmatrix} 0.71 \\ -0.71 \\ 0.00 \end{pmatrix}$	4.46	$\begin{pmatrix} -0.41 \\ -0.41 \\ 0.82 \end{pmatrix}$
		$I4/mcm$	5.51	$\begin{bmatrix} 4.07 & -0.82 & -1.31 \\ -0.82 & 4.07 & -1.31 \\ -1.31 & -1.31 & 4.25 \end{bmatrix}$	1.83	$\begin{pmatrix} 0.56 \\ 0.56 \\ 0.61 \end{pmatrix}$	4.90	$\begin{pmatrix} 0.71 \\ -0.71 \\ 0.00 \end{pmatrix}$	5.67	$\begin{pmatrix} -0.43 \\ -0.43 \\ 0.79 \end{pmatrix}$

Table S3. Bulk compressibility, β, elastic compliance matrix, $\boldsymbol{s}$ and eigenvalues, $s_L/s_M/s_H$, and corresponding normalised eigenvectors, $\hat{s}_L/\hat{s}_M/\hat{s}_H$, of $\boldsymbol{s}$ computed by DFT for all structures. All values given with units of TPa^{-1}.

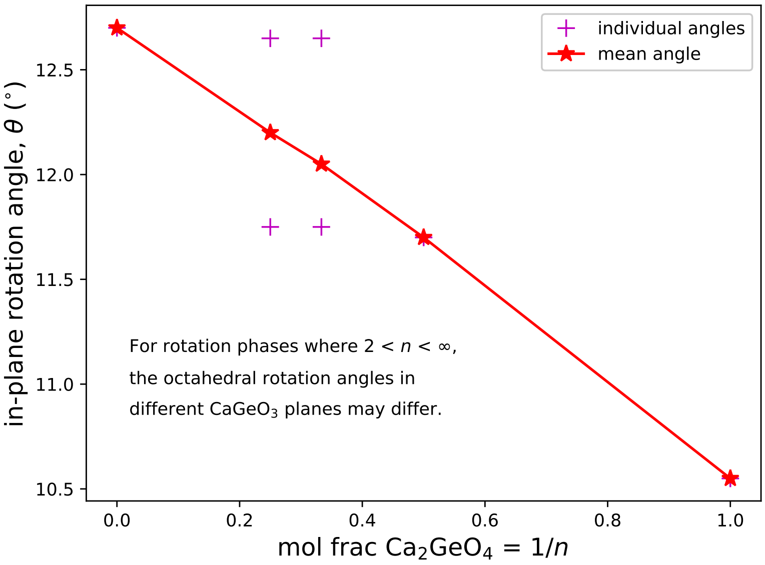

Figure S2. Frozen octahedral rotation angle about c axis in fully relaxed rotation phases as a function of $1/n$.
