# Supplementary material for: Control of Uniaxial Negative Thermal Expansion in Layered Perovskites by Tuning Layer Thickness
Source: Front Chem. 2018 Oct 18;6:455. doi: 10.3389/fchem.2018.00455 (PMC6201142; doi:10.3389/fchem.2018.00455)
Supplement: Supplementary file 2 [file Data_Sheet_2.pdf]

Appendices: Control of Uniaxial Negative Thermal Expansion in Layered Perovskites by Tuning Layer Thickness

APPENDIX 1 BACKGROUND THEORY

This section briefly summarizes some of the background theory relevant for understanding thermal expansion in an anisotropic material. An interested reader can find many more thorough explanations of these concepts in other sources (Ashcroft and Mermin (1976), Dove (1993)).

The elastic compliance matrix, $\boldsymbol{s}$, relates the anisotropic strain response of a material, $\boldsymbol{\varepsilon}$ to an applied stress, $\boldsymbol{\sigma}$, via the equation

$$\varepsilon_i = \sum_j s_{ij}\, \sigma_j, \tag{A1}$$

where ε_i (σ_i) is component i of $\boldsymbol{\varepsilon}$ ($\boldsymbol{\sigma}$) and $\boldsymbol{\varepsilon}$ ($\boldsymbol{\sigma}$) is a 6-dimensional vector expressed in Voigt notation so that the first three components ($i = 1, 2, 3$) describe normal strains (stresses) of the crystal and the latter three components ($i = 4, 5, 6$) describe shear strains (stresses). $\boldsymbol{s}$ is therefore a 6×6 matrix.

Using this definition of $\boldsymbol{s}$, the general anisotropic thermal expansion, $\boldsymbol{\alpha}$, of a material is given by the equation

$$\boldsymbol{\alpha} = \boldsymbol{s}\, \boldsymbol{\Phi}, \tag{A2}$$

where $\boldsymbol{\alpha}$ is the anisotropic thermal expansion vector expressed in Voigt notation, in response to an anisotropic driving force for thermal expansion, that we express by the vector $\boldsymbol{\Phi}$.

In a tetragonal material, the a and b axes are equivalent, and thus in the compliance matrix $s_{11} = s_{22}$ and $s_{13} = s_{12}$. Furthermore, all normal-shear coupling terms, s_{ij} ($i = 1, 2, 3$; $j = 4, 5, 6$), are 0 by symmetry and thus shear components of $\boldsymbol{\Phi}$ may not contribute to normal components of $\boldsymbol{\alpha}$. If we assume that we have a tetragonal material that remains tetragonal, and thus undergoes no shear deformations, Equation A2 simplifies to:

$$\begin{pmatrix} \alpha_1(T) \\ \alpha_1(T) \\ \alpha_3(T) \end{pmatrix} = \begin{pmatrix} s_{11} & s_{12} & s_{13} \\ s_{12} & s_{22} & s_{13} \\ s_{13} & s_{13} & s_{33} \end{pmatrix} \begin{pmatrix} \Phi_1(T) \\ \Phi_1(T) \\ \Phi_3(T) \end{pmatrix}. \tag{A3}$$

In Equation A3 we assume that $\boldsymbol{s}$ is temperature independent to a first approximation and therefore the temperature dependence of $\boldsymbol{\alpha}$ is given by the driving force for thermal expansion $\boldsymbol{\Phi}(T)$. We may then express $\boldsymbol{\Phi}(T)$ in terms of mode specific heat capacities, $C_v^i(T)$, and anisotropic mode Grüneisen parameters, $\boldsymbol{\gamma}^i$, by the equation

$$\Phi_\eta(T) = \sum_i C_v^i(T)\,\gamma_\eta^i. \tag{A4}$$

In Equation A4 the summation over indices i is really of every discrete phonon mode at every phonon wavevector on a sufficiently dense grid to approximate an integral over the Brillouin zone. The specific heat capacity of mode i is a function of the frequency of that mode, ω^i, and temperature T,

$$C_v^i = \frac{\hbar w^i}{V}\frac{\partial}{\partial T}\left[\left(e^{\frac{\hbar w^i}{k_B T}} - 1\right)^{-1}\right], \tag{A5}$$

where the derivative describes how the population of that mode increases with increasing T. The component γ_η^i of the vector $\boldsymbol{\gamma}^i$ then describes the contribution of mode i to thermal expansion of lattice parameter η, and is defined as

$$\gamma_\eta^i = -\frac{\partial \ln\left[w^i\right]}{\partial \ln\left[\eta\right]}, \tag{A6}$$

such that if $\gamma_\eta^i > 0$ mode i contributes to PTE of η and likewise if $\gamma_\eta^i < 0$ mode i contributes to NTE.

Equation A3 implies that even a $\boldsymbol{\Phi}$ vector with all positive components could be transformed into a uniaxial or biaxial NTE regime ($\boldsymbol{\alpha}$ with one or two negative components respectively) by a sufficiently anisotropic compliance matrix. This scenario is illustrated for a tetragonal material in Figure 2 in the main manuscript where a $\boldsymbol{\Phi}$ driving bulk PTE is transformed by a highly anisotropic $\boldsymbol{s}$ into the quadrant corresponding to uniaxial NTE of the c axis.

The degree of anisotropy can be quantified by the ratio, κ, of the highest and lowest eigenvalues of $\boldsymbol{s}$, s_H and s_L respectively, given as

$$\kappa = \frac{s_H}{s_L}. \tag{A7}$$

If $\kappa = 1$, the quadratic form of $\boldsymbol{s}$ in Figure 2 would be a sphere and $\boldsymbol{s}$ would not alter the direction of the vector $\boldsymbol{\Phi}$ in Equation A2. However, as κ becomes greater, the quadratic form of $\boldsymbol{s}$ becomes more ellipsoidal and thus $\boldsymbol{s}$ has the potential to rotate the direction of $\boldsymbol{\Phi}$. κ is thus a good metric to consider the potential for $\boldsymbol{s}$ to transform $\boldsymbol{\Phi}$ driving bulk PTE into α corresponding to anisotropic NTE.

APPENDIX 2 PHASE DIAGRAM AND SYMMETRY OF RUDDLESDEN–POPPER PHASES

In Figure 1 in the main manuscript, members of the $A_{n+1}B_nO_{3n+1}$ Ruddlesden–Popper series were displayed in the high-symmetry $I4/mmm$ parent structure. Figure A1 shows the phase diagrams relevant for NTE in the $n = 1$ and $n = 2$ systems. In Ca_2MnO_4 and $Ca_3Mn_2O_7$, the uniaxial NTE phase has an anti-phase frozen octahedral rotation about the c axis, corresponding to the $I4_1/acd$ or $Acaa$ space groups respectively. In $n = 1$ $I4_1/acd$, this rotation is anti-phase between adjacent equivalent BO_6 perovskite layers in different unit cells – the corresponding distortion is at $P = (1/2, 1/2, 1/2)$ – whereas in $n = 2$ $Acaa$ the rotations are anti-phase within each BO_6 block but with no doubling of the $I4/mmm$ unit cell along

c – corresponding to a distortion at $X = (½, ½, 0)$. In both systems, this NTE phase with anti-phase rotations competes with a *ground-state* phase with both frozen rotations (about c) and tilts (with rotation axes in the layering plane) of BO_6 octahedra, that is a child of an alternative rotation phase with in-phase rotations. The *ground-state* phase shown is found to be the lowest energy structure computed using DFT in $Ca_{n+1}Ge_nO_{3n+1}$. The analogous phase diagram for an ABO_3 perovskite (the $n = \infty$ RP end-member) is also shown for comparison, even though ABO_3 perovskites typically do not exhibit uniaxial NTE in their $I4/mcm$ phase with anti-phase rotations.

We previously used the concept of *symmetry trapping* to explain the presence of soft (low frequency), yet stable (real phonon frequencies), octahedral tilts driving uniaxial NTE in $n = 2$ $Ca_3Mn_2O_7$ (Senn et al. (2015)). This idea stems from the fact in the $n = 1, 2$ phase diagrams, that the crystal cannot transform from the NTE metastable phase to the ground-state phase without the frozen octahedral rotations changing sense – anti-phase rotations about c need to "unwind" to form the in-phase rotations in the ground-state phase. Thus soft phonons are able to persist without the structure undergoing a soft-mode phase transition to the ground state. In the ABO_3 perovskite, the c axis is not as strongly defined as in layered RP compounds (since there is no inherent layering topologically distinguishing a particular axis in the ABO_3 structure). Therefore, although there is no group-subgroup relationship between $I4/mcm$ and the ground-state $Pnma$ phases, there is no clear distinction between rotations and tilts and a phase transition with a relatively low activation barrier corresponding to a rotation of the direction of the out-of-phase octahedral tilting can be envisaged.

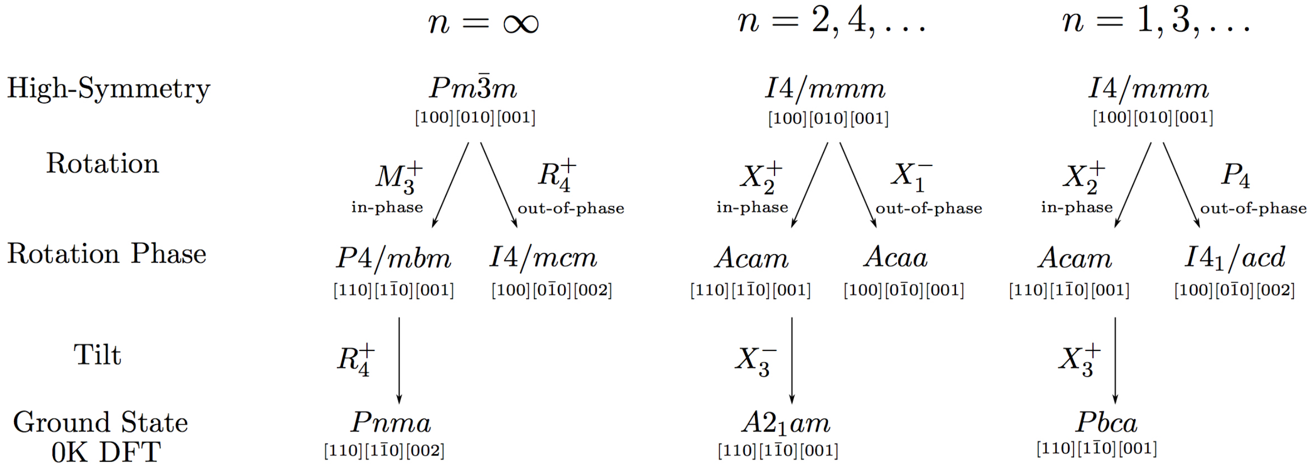

Figure A1. Space-group diagrams showing the relevant phases for uniaxial NTE in low n $A_{n+1}B_nO_{3n+1}$ Ruddlesden–Popper systems. The NTE phase has a frozen rotation of BO_6 octahedra about the layering axis, which is out-of-phase between adjacent unit cells (P_4 irrep) if $n = 1$ and out-of-phase with each perovskite block but in-phase between adjacent unit cells (X_1^- irrep) in the $n = 2$ system. This NTE phase competes with a ground state phase that has an in-phase frozen octahedral rotation about the layering axis and frozen octahedral tilt in the plane of the layering axis. For higher n, the $n = 1$ picture extends to odd values of n and the $n = 2$ to even values of n. An analogous phase diagram for the $n = \infty$ extreme of an ABO_3 perovskite is also shown even though the $I4/mcm$ phase seldom exhibits NTE.

The $n = 1$ phase diagram may be extended to all odd n in the Ruddlesden–Popper series and the $n = 2$ phase diagram to all even n. In this work, we distinguish between high-symmetry phases – the $I4/mmm$ RP or $Pm\bar{3}m$ perovskite parents – and rotation phases – by which we mean the NTE (or equivalent) phase

with anti-phase rotations about the c axis – in first-principles simulations of the $Ca_{n+1}Ge_nO_{3n+1}$ system ($n = 1, 2, 3, 4, \infty$).

Although the $Acam$ and $Acaa$ rotation phases have orthorhombic space groups, in DFT simulations in this work and from experimental measurement in previous works (Senn et al. (2015)) we in fact find them to be pseudo-tetragonal. This is because locally each BO_6 octahedron has 4-fold rotational symmetry and the frozen rotation angle is the same for octahedra in equivalent layer positions in different perovskite blocks in the unit cell, even if the sense (clockwise vs anticlockwise) of the rotation is different. This pseudo-tetragonality means that even though structures were relaxed in orthorhombic space groups with no additional symmetry constraints, the a and b lattice parameters are always found to be equal to within the accuracy of the simulation with no spontaneous in-plane distortion of the BO_6 units.

In the $n = 1, 3$ compounds, the structures (lattice parameters, relaxed cell energies, octahedral rotation angles) of the $Acam$ and $I4_1/acd$ phases were exactly equal (to the accuracy of the calculation), indicating that equivalent GeO_6 octahedra in adjacent $I4/mmm$ unit cells are sufficiently de-phased that the relative sense of their rotations has no effect on the structural properties of the crystal. Noting that in $n = 3$ $Acam$ the octahedral rotations are anti-phase within each perovskite block, exactly as in relaxed $I4_1/acd$ (although still in-phase between unit cells), we used $n = 3$ $Acam$ as a proxy for $I4_1/acd$ in calculations of elastic properties in sections 3.1–3.3 since many repeated cell relaxations were required that would have been extremely computationally expensive in the large $n = 3$ $I4_1/acd$ unit cell. However, for lattice dynamics calculations, $n = 3$ $I4_1/acd$ was studied since the phase of rotations between unit cells was found to give different frequencies for the softest tilt modes between $Acam$ and $I4_1/acd$ in the lattice dynamical calculations performed in section 3.4.

REFERENCES

Ashcroft, N. W. and Mermin, N. D. (1976). *Solid State Physics* (Belmont, CA, USA: Brooks/Cole)

Dove, M. T. (1993). *Introduction to Lattice Dynamics* (Cambridge, UK: Cambridge University Press)

Senn, M. S., Bombardi, A., Murray, C. A., Vecchini, C., Scherillo, A., Lui, X., et al. (2015). Negative thermal expansion in hybrid improper ferroelectric ruddlesden-popper perovskites by symmetry trapping. *Phys. Rev. Lett.* 114, 035701. doi:10.1103/PhysRevLett.114.035701
